# Examining the Relationship Between Assertiveness and Anxiety in First- and Second-Year US Medical Students

**DOI:** 10.2196/73394

**Published:** 2025-06-20

**Authors:** Jonathan Shaw, James Hagerty, Kristen Masada, Angelene Eunji Won, Ashley Lai, Jisu Shin, Van Le, Brenton Phung, Charles Lai, Peter Bota, Aaron Jacobs

**Affiliations:** 1 School of Medicine, California University of Science and Medicine, 1501 Violet St, Colton, 92324, United States, 1 9094980036; 2 Medical Education, California University of Science and Medicine, United States

**Keywords:** assertiveness, anxiety, medical student, preclinical, medical education, well-being

## Abstract

In this single-institution remote survey study of a California medical school, anxiety and assertiveness were found to inversely correlate in preclinical medical students.

## Introduction

Confident and assertive physicians’ development is essential to effective patient care [1]. Medical training shapes students’ knowledge, skills, and interpersonal abilities, including confidence and assertiveness, which evolve throughout education [2]. Assertiveness influences clinical decision-making, patient communication, and professional success [3]. Further, assertiveness has been shown to improve feelings of anxiety, stress, and depression [4]. Although literature confirms that training can improve assertiveness, factors that affect assertiveness and its gender-based differences have been underexplored in medical students, who face uniquely stressful hierarchical environments [1]. A better understanding of assertiveness and anxiety correlations can help educators tailor interventions to support students’ well-being and competence. To guide future curricular improvements, we investigate this relationship through the Simple Rathus Assertiveness Scale-Short Form (SRAS-SF) and the General Anxiety Disorder 7-item (GAD-7) assessment.

## Methods

### Participants and Recruitment

First-year and second-year medical students (n=120 and n=126, respectively) from a California allopathic school with a pass/fail curriculum completed an anonymous Google Forms survey from January 31 to February 29, 2024 (invited via institutional email). Submission of multiple responses was prevented via Google Forms’ single-response function. For study inclusion, students had to be in their preclinical years of the school’s Doctor of Medicine program; else, they were excluded. A convenience sample of 30 responses (23 and 7 responses from first-year and second-year medical students, respectively) was collected (Table 1).

**Table 1.** Descriptive statistics (responses: N=30).

Survey items	Value
Demographics, n (%)	
Which school year are you?	
First year	23 (77)
Second year	7 (23)
What gender do you identify as?	
Male	13 (43)
Female	17 (57)
SRAS-SF^a^^,^^b^ score, median (IQR)	
When I am eating out and the food I am served is not cooked the way I like it, I complain to the person serving it	−2.00 (−2.25 to 1.00)
There are times when I look for a good strong argument	1.00 (−2.00 to 2.00)
I try as hard in life to get ahead as most people like me do	1.00 (0.50 to 2.00)
If a famous person were talking in a crowd and I thought he/she was wrong, I would get up and say what I thought	−2.00 (−3.00 to −1.00)
If someone has been telling false and bad stories about me, I see him or her as soon as possible to “have a talk” about it	1.00 (−2.00 to 2.00)
I complain about poor service when I am eating out or in other places	−2.00 (−2.00 to −1.00)
If a couple near me in the theater were talking rather loudly, I would ask them to be quite or to go somewhere else and talk	−1.00 (−2.25 to 1.00)
I am quick to say what I think	0 (−1.00 to 1.00)
Most people stand up for themselves more than I do	0 (−2.00 to 2.00)
At times I have not made or gone on dates because of my shyness	−1.00 (−2.25 to 1.00)
If a person serving in a store has gone to a lot of trouble to show me something which I do not really like, I have a hard time saying, “No.”	−1.00 (−2.00 to 1.25)
To be honest, people often get the better of me	−1.00 (−2.00 to 1.00)
I do not like making phone calls to businesses or companies	1.50 (0.25 to 2.00)
I feel silly if I return things I don’t like to the store that I bought them from	−1.50 (−2.00 to 1.00)
If a close relative that I like was upsetting me, I would hide my feelings rather than say that I was upset	−1.00 (−1.25 to 2.00)
I have sometimes not asked questions for the fear of sounding stupid	2.00 (−1.25 to 2.25)
During an argument, I am sometimes afraid that I will get so upset that I will shake all over	−2.00 (−3.00 to −0.50)
I often have a hard time saying, “No.”	1.00 (−2.00 to 2.00)
When someone says I have done well, I sometimes just don’t know what to say	1.00 (−2.00 to 1.25)
SRAS-SF total score	−0.11 (−0.76 to 0.84)
GAD-7^c^^,^^d^ assessment score, median (IQR)	
Feeling nervous, anxious, or on edge	1.00 (0.75 to 2)
Not being able to stop or control worrying	0 (0 to 1.00)
Worrying too much about different things	1.00 (0 to 2.00)
Trouble relaxing	1.00 (0 to 2.00)
Being so restless that it’s hard to sit still	0 (0 to 1.00)
Becoming easily annoyed or irritable	1.00 (0 to 2.00)
Feeling afraid as if something awful might happen	0.50 (0 to 1.00)
GAD-7 total score	4.50 (2.00 to 11.25)

a SRAS-SF: Simple Rathus Assertiveness Scale-Short Form.

b SRAS-SF scoring: The SRAS-SF consists of 19 statements that participants indicate their agreement with, using a 6-point Likert-scale (−3=“very much unlike me”; 3=“very much like me”). Responses are averaged, resulting in total scores between −3 (less assertive) and 3 (more assertive).

c GAD-7: General Anxiety Disorder 7-item.

d GAD-7 scoring: The GAD-7 consists of 7 statements about anxiety symptoms, with participants indicating how often they experienced these symptoms within the last 2 weeks by using a 4-point Likert scale (0=“not at all”; 3=“nearly every day”). These scores are added together to determine anxiety severity: 0‐4 (minimal), 5‐9 (mild), 10‐14 (moderate), and 15‐21 (severe anxiety).

### Measures

The survey included demographic questions (school year and gender), 19 SRAS-SF items [5], and the GAD-7. The SRAS-SF and GAD-7 were presented in separate, randomized sections.

### Statistical Analysis

IBM SPSS Statistics 28.0.1.0 (IBM Corp) was used for analysis. Due to the small sample size (n=30), a Shapiro-Wilk test was used to assess if data were normally distributed [6]. Parametric (2-tailed independent samples *t* test and Pearson correlation) and nonparametric (Kruskal-Wallis test and Spearman correlation) statistical tests were used based on data distribution normality. Gender and school year were used as grouping variables.

### Ethical Considerations

This study received ethical approval from the California University of Science and Medicine Institutional Review Board (approval: HS-2024‐03) on January 22, 2024. Informed consent for primary data collection and secondary analyses of the data was obtained from all participants. Participants received no compensation for participation.

## Results

The Shapiro-Wilk test indicated that SRAS-SF scores (*P*=.07) were normally distributed, while GAD-7 scores (*P*=.01) and all individual survey items (*P*<.05) were not normally distributed.

Per the Kruskal-Wallis test for examining differences between responses by school year and gender, first-year medical students were more likely to feel uncomfortable when returning purchases (*P*=.03), and female participants were more likely to ask loud theater couples to be quiet (*P*=.05). No differences in GAD-7 scores by school year (*P*=.67) or gender (*P*=.52) were noted.

As the overall SRAS-SF scores were normally distributed, an independent *t* test was used; it found no significant differences in SRAS-SF scores by school year (*P*=.95) or gender (*P*=.62).

**Figure 1.**
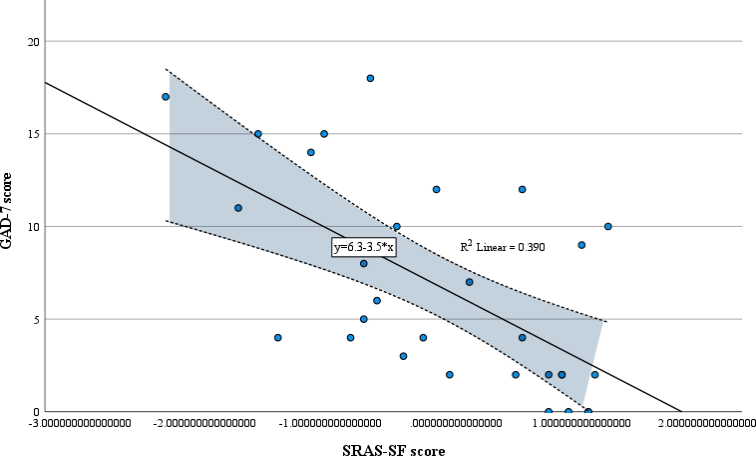
Scatterplot of GAD-7 scores by SRAS-SF scores. This figure was generated by using SPSS, and it visualizes the line of best fit between the GAD-7 and SRAS-SF scores. The *R*^2^ value is 0.39, and the *r* value is −0.62, indicating a strong negative correlation between GAD-7 and SRAS-SF scores. GAD-7: General Anxiety Disorder 7-item; SRAS-SF: Simple Rathus Assertiveness Scale-Short Form.

A Pearson correlation revealed a strong negative correlation between SRAS-SF and GAD-7 scores (n=30, *r*=−0.624; *P*<.001; Figure 1).

## Discussion

### Principal Findings

The differences between first-year and second-year medical students’ confidence toward returning purchases may reflect cohort-based personality variation. Female participants’ greater likelihood of addressing disruptive behavior may reflect gender differences in assertiveness, warranting further study on socialization and professional behaviors in medical education.

The strong negative correlation between assertiveness and anxiety aligns with research indicating medical students’ high anxiety levels [7], raising the possibility that greater assertiveness is linked to lower anxiety, though causality cannot be inferred [8]. To determine if assertiveness development mitigates anxiety, future studies should explore whether interventions targeting assertiveness influence students’ well-being. Given anxiety’s impact on academic performance and mental health, tailored strategies could help students in their training.

This study focuses on preclinical students, limiting applicability to clinical training or residency students. However, medical students face increasing anxiety due to high-stakes evaluations and residency match competitiveness [9,10]. Larger longitudinal studies could clarify this relationship and better inform future interventions.

### Limitations

Our small, single-institution sample limits generalizability and statistical power. Future studies should include multiple institutions to account for educational and cultural variations. Additionally, this study focuses on preclinical students, limiting relevance to clinical training or residency students.

### Conclusions

Our findings support the existing literature and suggest that assertiveness is inversely associated with preclinical medical students’ anxiety [8]. Although confidence typically improves with training, faculty and administrators can implement proactive strategies and training to support students’ interpersonal and professional development. Future research should explore longitudinal trends to refine educational interventions that enhance assertiveness and mental well-being.

## Abbreviations

GAD-7: General Anxiety Disorder 7-item
SRAS-SF: Simple Rathus Assertiveness Scale-Short Form
